# Chloroform

**Published:** 1865-04

**Authors:** M. M. Eaton

**Affiliations:** Peoria, Ill.


### CHLOROFORM.

By M. M. EATON, M. D., of Peoria, Ill.

This is one of the greatest blessings God has given to man, and if, by this short article I can attract the attention of a few of my Medical friends, and thereby influence them to use this most valuable remedy more frequently and more intelligently, I shall be doing my duty to the suffering sons and daughters of Eve.

I am well aware that there are a few timid practitioners who have sought to throw this remedy into disrepute, and there are a few careless or ignorant ones, who have partially succeeded, by means of their failures and accidents. Now I do not desire to be understood as saying that this remedy can do no injury. If this were so, then it is inert; for any good, active remedy must do injury when improperly used, or used in improper cases, or under improper circumstances. But what I do say is this, that most of the deaths that have occurred from Chloroform (so said), have been caused either from improper administration, improper circumstances, or when given to improper patients.

Now, thanks to Drs. Brainard and Miller, (who first taught me its proper administration, while under their tuition in Chicago City Hospital,) I can say, after a considerably extended practice in Surgery and Obstetrics, I have never had the least unpleasant effect from its administration, though using it almost daily the past five or six years.

1st. How should it be given?

2d. For what should it be given?

3d. To whom should it be given?

4th. What circumstances should be observed, and what precautions taken?

It should be inhaled in such a manner as to be freely mixed with the atmosphere. Simply pour a dram or two on a napkin, and hold within an inch or two of the nostrils, directing the patient to inhale freely—use none of the patent inhaling apparatus, they are more of a nuisance than a convenience—don't be alarmed if the patient is restless and talks incoherently, but give more until he will answer no more questions, keeping your finger on the pulse to see that it beats well.

So much for its administration; and here let me say I have never met a patient that could not be pleasantly influenced by this drug if it was given in large quantities enough. Now for the second question.

It should be given when any serious surgical operation is to be performed, excepting those in the mouth or throat where there will probably be great loss of blood, as in such cases there is danger of the blood choking the patient, if he is insensible. In other cases I deem it a cruelty to refuse to our suffering patient this invaluable boon. It should be given to women in labor when much desired always, and should be given anyhow where there is present a rigid, undilatable os uteri, and consequently a protracted labor. I often have in this way secured a speedy delivery in these cases, and have never had injury to result to mother or child from its use. Before turning I would always give it, unless my patient was so low from loss of blood as to be insensible, and as a consequence the parts thoroughly relaxed. It may be given to babes and young children fearlessly, when operating for Talipes, or any other painful operation. Your patient is then entirely under your control, and can be treated more skillfully and rapidly than could otherwise be possible; and the cries of the little patient will not unstring your nerves and make you incompetent to perform your task. Operating often for Talipes, I have always used Chloroform with great pleasure and satisfaction. For the convulsions of children, it is the most valuable remedy in the "Materia Medica." I cause the child in such cases to inhale the Chloroform till quiet sleep is induced, then sit by and watch it, and if convulsions again come on, give more Chloroform, and so continue till they disappear, then attend to the teeth, lance the gums, if necessary give a little soda, with or without calomel, as the case seems to require. This is the only treatment I have confidence in or would dare rely upon, as I have been very successful with it, and very unsuccessful with any other management.

It has gained favor with the majority of medical men that Chloroform should not ever be given to patients affected with Phthisis Pulmonalis, or Cardiac Disease. I have given it in both classes of patients without injury, and my experience in this is the same as that of Prof. Wright, of Cincinnati, related to me the past winter while visiting him. Therefore, I say it may be given to all patients requiring its use, if given properly and under proper circumstances.

What are these circumstances? First, have your patient in a recumbent posture—don't forget it. *Never give Chloroform to a patient sitting, standing, or semi-recumbent.* Dentists please note. Most of the accidents from Chloroform have been under your administration, where your patient was semi-recumbent. Always have the head nearly as low as the body. If syncope threatens, carry the head off the table or bed and hold it below the body, so as to allow the blood more readily to go to the brain and maintain life. Have the patient fast one meal before the Chloroform is to be given—much injury and annoyances often results from neglect of this precaution. If the patient is anæmic give a glass of brandy and water before the Chloroform. Have Spirits Ammonia by you, to be used if needed. Do not mix your Chloroform with Ether or anything else, if you want to be able to manage your case give *pure* Chloroform. This course has aided me materially in the little reputation I have in surgical and obstetrical practice. I hope to hear other physicians' experience with this valuable medicine.

DEATHS FROM CHLOROFORM.—In the *Medical Times and Gazette* of January 7th we find two more deaths from the use of chloroform reported, although special precautions were taken in administering it. The cases are published by Mr. J. Usher Huxley, House-surgeon to the Devon and Exeter Hospital. We copy the first in full, as follows:

"W. L., a healthy looking young man, aged 24, was admitted into the Devon and Exeter Hospital on Sept. 26th, suffering from a severe injury to the right foot. A heavy piece of timber falling on it had fractured the metacarpal bone of the great toe, and deeply lacerated the structures of the inner side and sole. The case went on well, and after the separation of some rather extensive sloughs, healthy granulations filled up the greater part of the chasm. The distal end of the fractured metacarpal bone, however, though evidently dead, remained fixed, and considerably hindered the progress of the wound. The man was therefore told that he must either be contented to remain in bed several weeks longer while the bone spontaneously separated, or must submit to a slight operation for its removal. He chose the latter, on condition that he might have chloroform. The heart was examined, and nothing being found to contra-indicate the employment of an anæsthetic, his wish was acceded to.

"On November 1, at 10 A. M., the patient was carried to the operating room. Two of the Hospital surgeons and the House-surgeon were present; three pupils and two nurses were also in attendance. Forty minims (measured) of chloroform were placed in a Snow's portable inhaler, and the inhalation was commenced, the external valve being at first turned aside and afterwards gradually closed. The man breathed calmly and showed no symptoms of annoyance or distress, and in about a minute began to smile, talk, and move his head slightly. The pulse, which was kept throughout, beat quite steadily for from two to three minutes, when it suddenly became imperceptible; there was no congestion or other change of the countenance, the pupils were not dilated, and the breathing was as yet quite natural; the inhaler, to which no fresh charge of chloroform had been added, was, however, immediately removed. In the course of a few seconds the respiration became deep and sobbing, and the tongue was protruded between the teeth; no return of the pulse could be felt. The only approach to spasm or convulsion was that for a few seconds the teeth were firmly clenched; this quickly passed off, and the tongue was again protruded.

The time during which the respirations continued, unaided, after the pulse had ceased, may be put down at fully a minute, and not until still later did the face become congested or the pupils dilated.

"The cold douche, ammonia, brandy, galvanism, and forceps for the tongue (as always at this Hospital) were at hand in the operating room. The first was applied directly the pulse was missed, and in the shortest space ot time brandy was injected into the rectum, solution of ammonia on lint was applied to the nostrils, and galvanic shocks were passed (with due intermission) through the cardiac region, whilst friction was applied to the extremities. The forceps were not needed, as the tongue did not fall back.

"Immediately the breathing began to flag, artificial respiration by the 'Sylvester method' was commenced, and this was steadily carried on for upwards of half an hour. The galvanism was applied at intervals for a like period. No trace of reaction occurred. It should be stated that the inhaler had not been used for any other case that morning, so that it contained absolutely no chloroform but the forty minims.

"*Autopsy six hours after death.*—Body muscular, well nourished. Throax—Pericardium natural; surfaces of right pleura everywhere firmly adherent; both lungs healthy in structure, the right more congested than the left. On removing the heart (all its orifices having been previously secured), the ventricles were found firmly contracted and empty; the auricles contained some blood, the right in larger proportion. In these and in the large veins the blood was mostly, but not wholly, uncoagulated. All the valves of the heart were perfectly healthy. The muscular substance was firm, of good color, and without the slightest appearance of fatty or other degeneration. On microscopical examination, the ultimate fibres were found free from oil-globules, and striæ were well marked. The walls of the left ventricle were slightly hypertrophied without dilatation. The whole organ, washed free from blood, but with a portion of the large vessels adhering, weighed 14½ ounces. Aorta healthy; no appearance of clots in the pulmonary artery. Abdomen—Stomach moderately filled with food. Spleen natural; weight 11 ozs. Liver large, congested; weight 5 lbs. 12 ozs. Kidneys, large and much congested; weight, the right, 9¼ ozs., the left, 9¾ ozs. Both liver and kidneys were firm to the knife, and the surface of the sections looked smooth and rather glistening, so that there was no question as to albumenoid (amyloid) degeneration; but there was no semi-transparent, glue-infiltrated appearance of the cut surfaces and edges, and no reaction, characteristic of the amyloid change, took place when solution of iodine was applied. Under the microscope, too, both the arteries and cells of the hepatic and renal structures appeared perfectly natural. After being allowed to remain for some hours (covered over. to prevent evaporation), it was found that a quantity of blood had drained from the cut surfaces, and that the liver had assumed the ordinary size, while the kidneys were much reduced in bulk. Sections now freshly made showed perfectly natural surfaces. Head (examined last)—The brain and its membranes were perfectly healthy, and not at all congested; blood, however, had probably drained away from the divided veins of the neck. Some urine drawn off from the bladder after the examination contained a trace of albumen, and a flocculent deposit consisting solely of epithelium.

"At the inquest held on this case the jury returned a verdict of 'Homicide by misadventure,' and wholly exculpated the medical officers from blame in the matter."

This case we look upon as one of the greatest value in its bearing on the question of the possibility of employing chloroform as an anæsthetic without danger. It could have been hardly possible to take greater precautions in administering it, or to use more faithfully all the means likely to have had any efficacy in the endeavor to rescue the patient from his perilous situation; and yet death followed. The eyes of the profession in Great Britain have been gradually opening to the alarming risk inseperable from the ordinary methods of inhaling this anæsthetic, and the recent report of the chloroform committee will undoubtedly have a good effect in checking its heedless employment, and in inducing greater watchfulness in those who are willing to deal with such a fatal agent. A new inhaler has been recommended, by which a proper dilution of the vapor with fresh air is secured, and the pulse is entrusted to a special guardian during the whole process of inhalation. With such precautions it is thought this anæsthetic may be disarmed of all its dangers. Now let us look at the case before us.

First, the patient was a young, healthy subject, presenting no constitutional contra-indication, so far as could be discovered, to the use of an anæsthetic. Second, the amount of chloroform inhaled was small—only forty minims—and given with great caution from an inhaler, with a due admixture of air with the vapor. Third, the pulse was watched from the first. And yet, without the slightest warning, without any evidence of distress or of cerebral oppression, without any faltering or weakness of the pulse to give the alarm, it instantly stopped, and did not return. Surely one who is accustomed to employ this agent may well be appalled by such an unlook-for casualty. Every known precaution taken, a fit subject for the inhalation, instant death without warning!

The case, as we have seen, is most carefully reported. The results of the autopsy are most interesting. It will be observed that the heart, *ultimum moriens* as it had been called, was found "firmly contracted and empty." This condition is very significant when considered in relation to the usually accepted explanation of chloroform deaths. Paralysis of the heart is the commonly received cause of death in these cases. The agent is thought to act with fatal rapidity by destroying the sensibility of the vital centre and thus arresting its action. It is pretty important matter to determine whether this is the true explanation of these terrible calamities, since upon it depends the theory of the treatment. It would seem, in the case before us, that death was caused by *spasm*, not paralysis, of the heart. The organ shut itself up so closely that not a drop of blood could enter it, and maintained its grip until the *rigor mortis* took its place. There were only slight signs of external spasm, the respiration was unobstructed and continued some time after the heart stopped beating, and yet it would not respond to the usual stimulus of the vital current. The blood was found to be mostly fluid. The poison had entered it and destroyed its normal properties, and the principal instrument in its distribution refused to drive it through the system. The deadly effect on the blood which chloroform produces cannot be too greatly emphasized. The mischief is most radical in its nature, and the entire suspension of the inhaling process cannot undo it in time to avert the danger. We are not aware that any investigations have been made to determine whether sulphuric ether produces any poisonous change in the blood when inhaled. Certain it is no instantaneous death has ever occurred under its use, and the symptoms which it causes diminish from the moment it is withdrawn from the patient.—*Boston Med. and Surg. Journal.*

